# Predicting the presence of depressive symptoms in the HIV-HCV co-infected population in Canada using supervised machine learning

**DOI:** 10.1186/s12874-022-01700-y

**Published:** 2022-08-12

**Authors:** Gayatri Marathe, Erica E. M. Moodie, Marie-Josée Brouillette, Joseph Cox, Curtis Cooper, Charlotte Lanièce Delaunay, Brian Conway, Mark Hull, Valérie Martel-Laferrière, Marie-Louise Vachon, Sharon Walmsley, Alexander Wong, Marina B. Klein, Canadian Co-Infection Cohort, Lisa Barrett, Jeff Cohen, Pierre Côté, John Gill, Shariq Haider, Neora Pick, Danielle Rouleau, Steve Sanche, Roger Sandre

**Affiliations:** 1 Department of Epidemiology, Biostatistics and Occupational Health, McGill University, 2001 McGill College Avenue, Montreal, QC H3A 1G1 Canada; 2 Centre for Outcomes Research and Evaluation, McGill University Health Center-Research Institute, Montreal, QC Canada; 3 Department of Psychiatry, McGill University, Montreal, QC Canada; 4 Department of Medicine, University of Ottawa, Ottawa, ON Canada; 5 Vancouver Infectious Diseases Centre, Vancouver, BC Canada; 6 Centre for Excellence, HIV/AIDS, St. Paul’s Hospital, Vancouver, BC Canada; 7 Department of Microbiology, Infectious Diseases and Immunology, Centre de Recherche du Centre Hospitalier de L, Université de Montréal, Montreal, QC Canada; 8 Centre Hospitalier de L’Université Laval, Quebec City, QC Canada; 9 Toronto General Hospital Research Institute, Toronto, ON Canada; 10 Department of Medicine, University of Saskatchewan, Regina, SK Canada; 11 CIHR Canadian HIV Trials Network (CTN), Vancouver, BC Canada

**Keywords:** HIV-HCV co-infection, Depressive symptoms, Supervised machine learning, Random forests

## Abstract

**Background:**

Depression is common in the human immunodeficiency virus (HIV)-hepatitis C virus (HCV) co-infected population. Demographic, behavioural, and clinical data collected in research settings may be of help in identifying those at risk for clinical depression. We aimed to predict the presence of depressive symptoms indicative of a risk of depression and identify important classification predictors using supervised machine learning.

**Methods:**

We used data from the Canadian Co-infection Cohort, a multicentre prospective cohort, and its associated sub-study on Food Security (FS). The Center for Epidemiologic Studies Depression Scale-10 (CES-D-10) was administered in the FS sub-study; participants were classified as being at risk for clinical depression if scores ≥ 10. We developed two random forest algorithms using the training data (80%) and tenfold cross validation to predict the CES-D-10 classes—1. Full algorithm with all candidate predictors (137 predictors) and 2. Reduced algorithm using a subset of predictors based on expert opinion (46 predictors). We evaluated the algorithm performances in the testing data using area under the receiver operating characteristic curves (AUC) and generated predictor importance plots.

**Results:**

We included 1,934 FS sub-study visits from 717 participants who were predominantly male (73%), white (76%), unemployed (73%), and high school educated (52%). At the first visit, median age was 49 years (IQR:43–54) and 53% reported presence of depressive symptoms with CES-D-10 scores ≥ 10. The full algorithm had an AUC of 0.82 (95% CI:0.78–0.86) and the reduced algorithm of 0.76 (95% CI:0.71–0.81). Employment, HIV clinical stage, revenue source, body mass index, and education were the five most important predictors.

**Conclusion:**

We developed a prediction algorithm that could be instrumental in identifying individuals at risk for depression in the HIV-HCV co-infected population in research settings. Development of such machine learning algorithms using research data with rich predictor information can be useful for retrospective analyses of unanswered questions regarding impact of depressive symptoms on clinical and patient-centred outcomes among vulnerable populations.

**Supplementary Information:**

The online version contains supplementary material available at 10.1186/s12874-022-01700-y.

## Background

With shared modes of transmission, co-infection of human immunodeficiency virus (HIV) and hepatitis C virus (HCV) is common, with approximately 2.3 million co-infected individuals worldwide [1, 2]. Depression is the most common neuropsychiatric manifestation among people living with HIV and those with chronic HCV. The prevalence of diagnosed clinical depression is two to fourfold higher among people living with HIV than the general population and reported to be as high as 24% among those with chronic HCV infection [3, 4]. Potential biological mechanisms include direct infection of the central nervous system and peripheral immune responses which have been shown to induce depression [3, 5]. Psychosocial risk factors including stigma, discrimination, lack of support, and substance use have also been shown to be contributory [3, 5]. Studies report an even higher depression prevalence in the co-infected population, which may be due to the co-existence of risk factors [6].

The presence of significant depressive symptoms may have an impact on outcomes in patients, even in the absence of a clinical depression diagnosis; for example, the presence of depressive symptoms is associated with non-adherence to antiretroviral therapy among people living with HIV, which may lead to increased viral load and suppressed immune function [7, 8]. Screening tools can be used to assess presence and severity of depressive symptoms and identify those at risk for major depression [9, 10], permitting early intervention. Co-infected individuals often live with multiple co-morbid conditions and thus spend a considerable amount of time in healthcare settings [11]. Depression screening among HIV and HCV infected patients is seldom routinely performed during clinical assessments or in longitudinal cohort studies, despite the known high prevalence of depression [12–14]. Multiple demographic, clinical and behavioural characteristics have been documented as risk factors for depression [15] and these data are generally collected in clinical and research cohorts. Thus, such data could be used retrospectively to predict the presence of depressive symptoms severe enough to be associated with negative health outcomes or an increased risk of being diagnosed with major depression and to follow this risk over time. Such measures will be useful for exploring important questions regarding depressive symptoms, their evolution and response to therapies in the co-infected population.

Machine learning includes robust techniques that enable accurate outcome predictions in medical research. In mental health research, machine learning has been used to predict current or future onset, disease course and treatment outcomes for psychiatric disorders including depression, anxiety, and schizophrenia [1, 16, 17]. A wide range of data sources have been used for developing these prediction algorithms including electronic medical records, neuroimaging, and social media. Demographic and clinical data have been used to create depression prediction models in the elderly and people with diabetes [18, 19]. However, similar models have not yet been developed in people living with HIV-HCV co-infection.

We leveraged a non-parametric supervised machine learning technique using cohort data to develop classification algorithms to predict the presence of depressive symptoms indicative of a risk for clinical depression and characteristics important for prediction of depressive symptoms in HIV-HCV co-infected individuals in Canada.

## Methods

### Data sources and study sample

We used data from the Canadian HIV-HCV Co-Infection Cohort (CCC), an open multicenter prospective cohort study, ongoing since 2003 and an associated sub-study, the Food Security and HIV-HCV co-infection study (FS sub-study) [20, 21]. The CCC recruits from 18 HIV centers, both urban and semi-urban across six Canadian provinces (Quebec, British Columbia, Alberta, Ontario, Nova Scotia, and Saskatchewan) [20]. Eligibility criteria include ≥ 16 years of age, documented HIV infection, and evidence of HCV infection (HCV RNA positive and/or HCV seropositive). The study had recruited 2018 participants as of July 2020. Participants are followed longitudinally, with follow-up visits every six months. Sociodemographic, behavioural, and health-related quality of life (HR-QoL) data are collected from participants by a standardized self-administered questionnaire at each visit. HR-QoL is measured using EuroQol-5 Dimension-3 Level (EQ-5D-3L) [22]. Clinical data including HIV/HCV treatment, co-morbidities, psychiatric diagnoses, and other medications are collected via medical chart reviews. Laboratory testing at each visit include HIV and HCV related tests, hematology, biochemistry, and liver profiles.

The FS sub-study is a mixed methods study conducted within the CCC between 2012 and 2015. All CCC participants were invited to participate and study visits were integrated into the biannual CCC visits. The FS sub-study recruited 725 participants and they were followed up for a maximum of 5 visits. The study collected data on food insecurity, general and mental health (including depression screening), treatment adherence and health care utilization using a self-administered questionnaire [21].

Depression screening was performed only in the FS sub-study; thus, the analytic sample in this study only included FS sub-study participant visits. The FS sub-study visits were merged with corresponding CCC visit data. As the two study visits for CCC and FS sub-study were, on occasion, not on the same day, information from visits within 3 months of each other were considered ‘concurrent’. We used three exclusion criteria to create the final study sample—1) participant visits were excluded if no depression screening measure (see below) was available at that visit; 2) participant visits were excluded if there was no corresponding CCC visit (within the 3-month window), as the predictors used in this analysis were derived from the CCC; and 3) all visits for a participant were excluded if no data was available for a predictor in all of their study visits.

### Outcome

Depression screening was conducted in the FS sub-study using the Center for Epidemiologic Studies Depression Scale-10 (CES-D-10), which is a shortened version of the CES-D-20 scale [23]. The CES-D-10 is a 10-item Likert scale questionnaire that assesses presence and severity of depressive symptoms in the past one week. Each item is measured on a 4-point scale, with reverse scoring for the 2 positive items and a total score range of 0–30. We dichotomized the score at 10 to create the CES-D-10 classes (1/0), as a score ≥ 10 is widely considered for the presence of depressive symptoms indicative of high risk for clinical depression, hereafter referred to as depressive symptoms for brevity [23]. Both the scale and the dichotomization at 10 have been validated in HIV populations in Canada [24].

### Predictors

We selected candidate predictors (x) from the CCC data based on the literature and subject matter expertise. We included predictors from five major categories: questions related to mental health, HR-QoL, sociodemographic, behavioural, and clinical characteristics; see Table 1. We selected a total of 137 candidate predictors, of which 136 were categorical and 1 was continuous (EQ-5D-3L—health state). From this list of candidate predictors, we selected a subset of predictors (x = 46) that may be more regularly available in most research settings based on expert opinion. See Table S1 in Appendix A for an exhaustive list of candidate predictors (x = 137) and their corresponding categories.

**Table 1:** Candidate predictors used in the random forest algorithms

Category	All Candidate Predictors
Questions related to mental health	Psychiatric institution or psychiatric hospital stay; Psychiatric diagnoses in chart reviews; Use of psychotropic medications (e.g., antidepressants, sedative hypnotics, atypical and typical anti-psychotics)
HR-QoL	EQ-5D-3L—standardized instrument - Descriptive system (mobility, self-care, usual activities, pain/discomfort, and anxiety/depression) - Current health state with a visual analog scale – scores range between 0–100
Sociodemographic characteristics	Age; Gender; Race/ethnicity; Immigration status; Living situation; Shared accommodation details; Education; Employment; Monthly income; Source of income
Behavioral characteristics	Injection drug use (ever/P6M); Non-injection drug use (ever/P6M); Needle/equipment sharing behaviors (ever/P6M); Snort (ever/P6M); Sharing behaviors for snorting apparatus (ever/P6M); Marijuana use; Therapy for drug addiction; Alcohol use (ever/P6M); Alcohol abuse; Smoking (ever/P6M); Sexual orientation; number of sexual partners; Sex work; Incarceration (ever/P6M); Tattoos; Body piercing
Clinical characteristics	BMI category; HIV viral load; CD4 count; HCV RNA; HIV disease stage; AIDS defining illness; health services used in the past 6 months (walk-in clinic, emergency room, inpatient, general practitioner, HIV clinic and specialist); Previous interferon-based HCV treatment; current antiretroviral therapy; Hepatitis B diagnosis; sexually transmitted disease diagnosis; end-stage liver disease (cirrhosis, ascites, varices, portal hypertension, encephalopathy, hepatocellular carcinoma); APRI, a measure of liver fibrosis; cardiovascular disease; autoimmune disease; hypertension; thyroid disease; psoriasis; lipodystrophy; hypercholesterolemia

*Abbreviations*: *HR-QoL* Health related quality of life, *EQ-5D-3L* EuroQoL-5Dimension-3Level, *P6M* in the past 6 months, *BMI* Body Mass Index, *HIV* Human Immunodeficiency Virus, *CD4* Cluster of differentiation 4 receptor, *HCV* Hepatitis C virus, *RNA* Ribonucleic acid, *AIDS* Acquired Immunodeficiency Syndrome, *APRI* Aspartate Aminotransferase (AST) to platelet ratio

### Statistical analysis

#### Primary analysis

We assessed proportion of missing data for each predictor. For predictors with < 5% missing data, we carried the value from the last visit forward. For predictors with ≥ 5% missing data and when data were missing for a predictor for all visits for a participant, we used an additional category of “no response” for categorical variables, which we hypothesized could be informative in the prediction algorithm (See Table S1 in appendix A) since the participant decision to respond (or not) is itself potentially clinically informative.

We used the supervised machine learning technique of Random Forests (RF), an ensemble learning approach which uses bootstrap aggregation of multiple decision trees, combining predictions from these many trees [25]; see more details about RF in Appendix B. We used probability machines to estimate the CES-D-10 class probabilities at each visit and then determined CES-D-10 class at default probability threshold [26]. We developed two RF algorithms—1. Full algorithm: Using all candidate predictors (x = 137) and 2. Reduced algorithm: Using a selected subset of more commonly available predictors (x = 46) based on expert opinion, which could be more generalizable to research studies beyond the CCC; see appendix A. We split the analytical sample into training and testing data, using the recommended 80:20 split, such that we had data for performance evaluation (testing) that was completely independent of data used for model development (training) and thus ensure an unbiased evaluation [27]. We performed the 80/20 split using the “createdatapartition” function from the Caret package in R, such that both CES-D-10 classes were represented in each set [28]. The algorithm was then developed by tenfold cross-validation using only the training data and RF hyperparameters (i.e., various RF settings like number of decision trees) were tuned to maximize accuracy [27]; see Appendix C for details.

#### Additional analyses

We conducted several analyses to provide additional details about the classification characteristics from the main analysis and assess robustness of the results: A) Using one visit per individual (total 717 visits), to assess difference in performance compared to the use of multiple visits per individual; B) Algorithms using three different CES-D-10 thresholds—8, 13, and 15—based on suggested cut-offs in the literature [29, 30]; C) An algorithm that included food insecurity as a predictor, which was collected only in the FS sub-study. Food insecurity in the past 6 months was measured using a 10-item adult scale of the Household Food Security Survey Module (HFSSM) [31]. A categorical variable was used, with participants with 0–1, 2–5, or ≥ 6 affirmative responses classified respectively as being food secure, moderately food insecure, or severely food insecure, as per the Health Canada criteria; and D. RF regression algorithms to predict continuous CES-D-10 score, evaluated using R-squared and root mean squared error (RMSE), which provides information regarding differences between the predicted scores and the actual scores [32].

### Performance evaluation

The final tuned algorithms were implemented in the testing data, which was not used in the development stage. The tuning parameters are shown in Table S2 in Appendix C. The overall performance and calibration measures are described in detail in Appendix C. To assess the ability to distinguish between classes (discrimination), we plotted receiver operating characteristic (ROC) and estimated the area under the ROC curve (AUC) [32, 33]. We used the default probability threshold of 0.50 for classification and at this threshold, sensitivity, specificity, positive predictive value (PPV), negative predictive value (NPV), positive likelihood ratio (LR +) and negative likelihood ratio (LR-) measures were then estimated with the 95% confidence intervals (CI) [34]. Finally, the RF importance metrics were generated, and importance plots were generated to present the 25 most important predictors in classifying participants with depressive symptoms by the two algorithms. We used RStudio v.1.2 and Stata v.16.0 to develop and evaluate these algorithms [35, 36]. The development of the RF algorithms was done using R package *ranger and caret*; for performance evaluation, we used the performance assessment function in R by Wong et. al. (2019) [28, 37–39].

## Results

### Study population

Of the 1973 FS sub-study visits in a total of 725 participants, 39 study visits were excluded based on the exclusion criteria described in the methods—16 visits (2 participants) with no CES-D-10 score, 18 visits (5 participants) with no con-current CCC visit and all 5 visits from 1 participant with no predictor data (EQ-5D health state) in all visits. Thus, 717 participants with a total of 1934 visits contributed to the final study sample. The participant characteristics at the first visit included in the sample are described in Table 2. The median CES-D-10 score was 10 (IQR, 5, 15), with 53% of the participants reporting the presence of depressive symptoms with CES-D-10 scores ≥ 10; 45% were prescribed one or more psychotropic medications such as bupropion and citalopram at baseline, but only 10% had a diagnosis of depression documented in their medical chart. Participants were predominantly male (73%) and white (76%). The population was vulnerable in terms of socioeconomic status (SES) characteristics with 73% unemployed, 76% with monthly income < $1500, 52% with high school being the highest level of education and 46% receiving welfare at baseline. Approximately 34% were current injection drug users, 62% current alcohol drinkers and 75% were current tobacco smokers. Only a small proportion had advanced liver disease (4%) or a current AIDS related illness (4%) and 35% were asymptomatic with a current CD4 cell count > 500 cells/μl (CDC clinical staging—A1).

**Table 2:** Baseline characteristics of participants in the study sample (*n* = 717)

Characteristics	Participants (*n* = 717) n (%) or median (IQR)
Age	49 (43, 54)
Gender – Male	522 (73)
Race/Ethnicity	
Asian	11 (2)
Black	28 (4)
White	541 (76)
Metis	32 (5)
First nation	102 (14)
Hispanic/Latino	7 (1)
Born outside Canada	64 (9)
Education—High school educated	376 (52)
Employment—Unemployed	525 (73)
Monthly income—< $1500	543 (76)
Revenue Source—Welfare	332 (46)
Current injection drug use	244 (34)
Current alcohol use	444 (62)
Current smoking	534 (75)
BMI category	
Underweight (< 18.5 kg/m^2^)	40 (6)
Normal weight (18.5–25 kg/m^2^)	312 (44)
Overweight (25–29.9 kg/m^2^)	189 (26)
Obese (30.0 kg/m^2^)	87 (12)
End-stage Liver disease	27 (4)
HIV clinical stage—A1 (Asymptomatic and CD4 > 500 cells/μl)	248 (35)
Past AIDS related illness	28 (4)
CES-D-10 score	10 (5, 15)
CES-D-10 category—≥ 10	382 (53)
Depression diagnosis	68 (10)
Prescribed antidepressant medications	320 (45)
HR-QoL using EQ-5D-3L instrument	
Anxiety/depression	
Not anxious or depressed	352 (49)
Moderately anxious or depressed	302 (42)
Extremely anxious or depressed	60 (8)
Current health state (visual analog scale)	70 (56, 80)

*Abbreviations*: *IQR* Interquartile range, *CES-D-10* Center for Epidemiologic Studies Depression Scale-10, *AIDS* Acquired Immunodeficiency Syndrome, *HIV* Human Immunodeficiency Virus, *BMI* Body Mass Index, *HR-QoL* Health related quality of life, *EQ-5D-3L* EuroQoL-5Dimension-3Level

### Performance evaluation

**Fig. 1:**
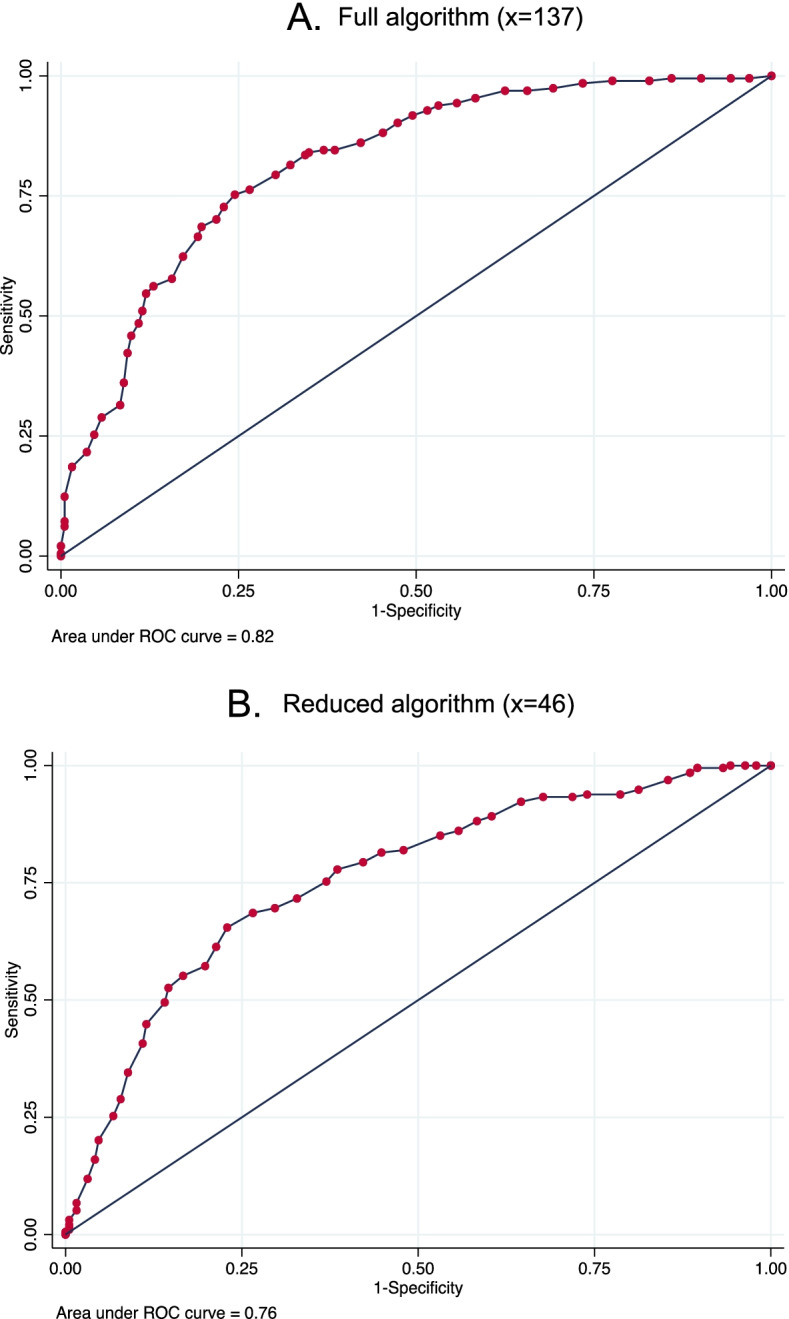
Receiver Operating Characteristic (ROC) curve for the **A** Full algorithm (x = 137) and **B** Reduced algorithm (x = 46)

**Fig. 2:**
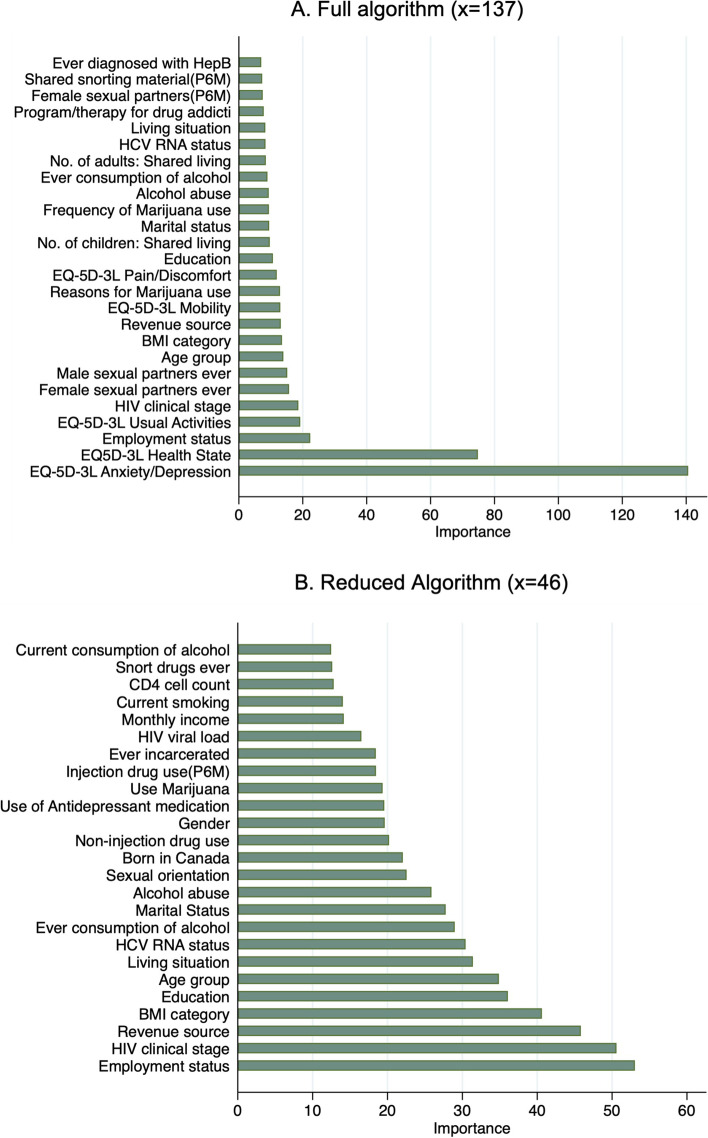
Predictor importance plots: **A** Full algorithm and **B** Reduced algorithm. Abbreviations: BMI: Body Mass Index; P6M: In the past 6 months; CD4: Cluster of differentiation 4 receptor; EQ-5D-3L: EuroQoL-5Dimension-3Level; RNA: Ribonucleic acid; Hep B: Hepatitis B virus

The training data consisted of 1548 visits and testing data consisted of 386 visits. The algorithms in the primary analysis showed acceptable calibration, as seen in Figure S1 in Appendix C. With regard to discrimination, the ROC curve for the primary analyses is shown in Fig. 1, with the curve close to the upper left-hand corner. The estimated AUCs wert 0.82 (95% CI: 0.78–0.86) and 0.76 (95% CI: 0.71–0.81) for the full and reduced algorithms respectively. The estimated sensitivity, specificity, PPV, NPV, LR + and LR- are presented in Table 3. The importance plots with 25 most important predictors for both algorithms are shown in Fig. 2. Employment, HIV clinical stage, revenue source, body mass index (BMI), and education were the 5 most important predictors.

**Table 3:** Performance evaluation in the primary analysis

Evaluation measure (95% CI)	Full algorithm (x = 137)	Reduced algorithm (x = 46)
AUC	0.82 (0.78–0.86)	0.76 (0.71–0.81)
Sensitivity	0.77 (0.70–83)	0.70 (0.63–0.76)
Specificity	0.73 (0.66–0.79)	0.70 (0.63–0.76)
PPV	0.74 (0.68–0.80)	0.70 (0.63–0.76)
NPV	0.76 (0.69–0.82)	0.69 (0.62–0.76)
LR +	2.8 (2.2–3.6)	2.3 (1.8–2.9)
LR -	0.3 (0.2 – 0.4)	0.4 (0.3–0.6)

Abbreviations: *CI* Confidence interval, *AUC* Area under the Receiver Operating Characteristic curve, *PPV* Positive Predictive Value, *NPV* Negative Predictive Value, *LR* + Positive likelihood ratio, *LR*—Negative likelihood ratio

The results for the additional analyses A-D are shown in Table 4 and summarized here. A) When using information from a single visit per individual, the overall performance was much lower, with AUC of 0.74 vs 0.82 for the full algorithm and 0.60 vs 0.76 for the reduced algorithm. B) For algorithms with the additional CES-D-10 thresholds, the AUC point estimate was higher for the full algorithm for cut-off 15 compared to 10 (0.87 vs 0.82). The other cut-off estimates were similar for to the corresponding algorithms for cut-off 10, with overlapping confidence intervals. C.) The algorithm including the additional predictor of food insecurity had a similar AUC estimate to the full algorithm, with overlapping confidence intervals. D.) The full algorithm predicting continuous CES-D-10 scores had a R-squared of 0.5 indicating that the algorithm explained only 50% of the variability in the CES-D-10 scores and had a high RMSE of 4.8, while for the reduced algorithm with a r-squared of 0.3, the algorithm explained only 30% of the variability in the scores and also had a high RMSE of 5.5.

**Table 4:** Comparison of performance evaluation measures for primary and additional analyses

Sr. No	Analysis	OOB error	AUC (95% CI)	Sensitivity (95% CI)	Specificity (95% CI)
**Primary Analyses**					
1	Full algorithm (x = 137)	0.16	0.82 (0.78–0.86)	0.77 (0.70–0.83)	0.73 (0.66–0.79)
2	Reduced algorithm (x = 46)	0.20	0.76 (0.71–0.81)	0.70 (0.63–0.76)	0.70 (0.63–0.76)
**Additional Analyses**					
**A**	**One visit per individual**				
1	Full algorithm (x = 137)	0.17	0.74 (0.66–0.82)	0.70 (0.58–0.80)	0.64 (0.52–0.76)
2	Reduced algorithm (x = 46)	0.23	0.60 (0.50–0.69)	0.73 (0.61–0.82)	0.37 (0.26–0.50)
**B**	**Different CES-D-10 cut-offs**				
**I**	**Cut-off – 8**				
1	Full algorithm (x = 137)	0.17	0.85 (0.81–0.89)	0.91 (0.87–0.95)	0.67 (0.59–0.74)
2	Reduced algorithm (x = 46)	0.19	0.79 (0.74–0.83)	0.83 (0.77–0.87)	0.60 (0.52–0.67)
**II**	**Cut-off – 13**				
1	Full algorithm (x = 137)	0.15	0.80 (0.76–0.85)	0.62 (0.54–0.70)	0.81 (0.76–0.86)
2	Reduced algorithm (x = 46)	0.20	0.79 (0.74–0.83)	0.45 (0.37–054)	0.87 (0.82–0.91)
**III**	**Cut-off—15**				
1	Full algorithm (x = 137)	0.14	0.87 (0.83–0.91)	0.55 (0.45–0.65)	0.95 (0.91–0.97)
2	Reduced algorithm (x = 46)	0.17	0.73 (0.67–0.79)	0.27 (0.19–0.36)	0.94 (0.91–0.97)
**C**	**With food insecurity variable (x = 138)**	0.16	0.84 (0.80–0.87)	0.77 (0.70–0.83)	0.73 (0.67–80)

Abbreviations: *OOB* Out-of-Bag Samples, *AUC* Area under the Receiver Operating Characteristic curve, *CI* Confidence interval, *CES-D-10* Center for Epidemiologic Studies Depression Scale-10

## Discussion

We developed a random forest algorithms using patient data from a cohort study that reliably predicted the presence of depressive symptoms indicative of a risk of clinical depression in a vulnerable HIV-HCV co-infected population. The algorithms used a set of selected candidate predictors (x = 137) from the cohort. The full algorithm using all candidate predictors performed better, with an AUC of 0.82, which indicates a 82% chance of distinguishing between CES-D-10 classes compared 0.76 for the reduced algorithm which used a smaller subset (x = 46) [33]. The prevalence of depressive symptoms was very high in our study, with more than 50% individuals found to be at risk for depression by CES-D-10 at their first visit. Despite this, only 10% had a documented depression diagnosis in their medical record suggesting there could be a substantial underestimation of the burden of depressive illness in this population without screening.

We developed this tool to assess if patient data that is commonly collected in clinical charts and research studies could be useful in predicting presence of depressive symptoms, which is seldom directly measured routinely for all patients, nor measured repeatedly over time. This tool will be most useful for conducting longitudinal clinical and epidemiologic research rather than for clinical care. It may prove useful to help identify people at risk for depression, study how this risk changes over time and with various interventions.

Most studies using machine learning have predicted the future onset of depressive symptoms [19, 40, 41] while a few, like ours, have focused on current depression prediction [42, 43]. A variety of predictors including demographic and clinical data, past medical history and life events have been studied. A range of machine learning algorithms like artificial neural networks, support vector machines, naïve Bayes classifier, and random forest were used in the general population and for specific groups like geriatric population and people with diabetes. These algorithms yielded AUC measures similar to ours, ranging between 0.70–0.95.

CCC collects extensive demographic, behavioral and clinical data. Using the full range and diversity of available predictors did show excellent discrimination in the full algorithm. However, for greater applicability, we chose to use a subset of 46 predictors that may be more readily available in other research settings, and despite using one third the number of predictors and less granular data, the overall discrimination was still acceptable. The additional analysis using only one visit per participant had a comparatively lower AUC, which may have been due to the smaller sample size and thus lower variability in the available data.

The algorithm we developed was a purely prediction algorithm and hence estimation of the strength of the effect of individual predictors is not possible. Further analysis with different modeling strategies would be needed for this assessment. However, the algorithm does provide some insight into factors that may be important for classification. The five most important predictors are related two main themes—i. SES (education, revenue source and employment) and ii. overall health status (HIV clinical stage and BMI). SES is a known strong determinant of depression. Receiving welfare and being from a low-income household has been associated with an elevated risk of food insecurity, and mental health issues [44, 45]. In Canada, almost 20% of people with major depression have been reported to be unemployed [46]. Another important health status related predictor was BMI. There have been studies with conflicting results regarding association between BMI and depression, and possible difference across race and gender [47, 48] and that BMI categories may not adequately capture people’s health status and thus this predictor needs to be considered with caution [49]. Finally, in the full algorithm with all 137 predictors, the EQ-5D-3L anxiety/depression dimension was the most important predictor and all EQ-5D dimensions (mobility, self-care, usual activities, pain/discomfort, and health state) were among the 25 most important. This provides further evidence that participant’s health status, and in the case of EQ-5D-3L, their perceived health status, are important in predicting depressive symptoms.

This study thus has many strengths. The CCC is generalizable to the HIV-HCV co-infected patients engaged in care in Canada, due to the recruitment from a variety of clinical settings (outreach, primary and tertiary care clinics in urban and semi-urban areas across the country). The sample used to develop these algorithms was generalizable to the parent CCC (see Appendix D; Table S3). The methodology used, RF, is non-parametric, highly accurate, and relatively robust to outliers, noise and does have safeguards from overfitting and thus improves chances of applicability beyond the data. Nevertheless, external validation is needed before application in other cohorts and research, to mitigate the risk of overfitting. In addition, the predictor importance plots provided some insight regarding predictors that play a major role in the accurate prediction of depressive symptoms.

The study however has limitations. The sample size is small as compared to big data applications of RF using electronic health records. Some predictors described in other studies such as childhood trauma, food insecurity among others, were not available for the full CCC. For example, in the additional analysis where we added the food security variable that was collected only in the food security sub-study was included, the AUC was slightly higher. Additionally, we categorized the CES-D-10 to create the binary classes, and thus may have lost some data by not predicting the individual CES-D-10 scores. We did develop a regression algorithm to predict the continuous CES-D-10 scores in additional analysis E, but it could only explain a small portion of the variability in the outcome. The gold standard depression diagnosis was not available in this study and thus the validity of the cut-off of 10 could not be assessed directly in this sample. In general, the overall AUCs were similar when using three other suggested CES-D- 10 cut-offs (8, 13, and 15) compared to a cut-off of 10. However, the full algorithm using a cut-off of 15 appears to have a higher AUC (0.87) than that of using a cut-off 10 (0.82). It will be important to assess in future studies whether this higher threshold may be more applicable to the co-infected population. However, since the CES-D-10 cut-off of 10 has been validated in HIV populations in Canada [24], we decided to use this threshold for comparability with available literature and future studies which may use this common threshold.

With a high proportion of participants with depressive symptoms in this population, it is important not to miss possible cases. Even if the algorithms we developed are considered to have acceptable discrimination (≥ 0.7) based on arbitrary thresholds, we would still misclassify a fair proportion of cases and thus this possible misclassification needs to be considered. Finally, this algorithm is applicable when the majority of the predictors are collected. However, in settings where such data is not available, especially completely clinical non-research setting implementing routine screening tools like the CES-D-10 should be considered, especially given the high prevalence of depressive symptoms we observed in this co-infected population.

## Conclusions

Depressive symptoms indicative of a risk for clinical depression were common in our population of people living with HIV-HCV co-infection. The random forest algorithms we developed shows promise in accurately predicting an elevated risk of clinical depression using data on patient characteristics collected in research settings. The algorithms identified important characteristics for depressive symptoms classification including employment, HIV clinical stage, revenue source, BMI, and education. Such machine learning algorithms can be used in research settings especially cohort studies where such data may be available to predict presence of depressive symptoms and use this information to understand the impact of depressive symptoms on clinical, health service and patient-reported outcomes in vulnerable populations.

## Supplementary Information

**Additional file 1.**

## Data Availability

The datasets generated and/or analysed during the current study are not publicly available. According to the stipulations of patient consent provided and our Institutional Ethics review boards, study records including confidential information collected during the study must be stored securely for 25 years after study completion, as required by Canadian clinical trial regulations. However, data stripped of personal identifiers, may be shared upon request to the corresponding author, or to: Mr. Sheldon Levy, Clinical Trials 2 Research Ethics Board (REB) Coordinator, MUHC Centre for Applied Ethics (sheldon.levy@muhc.mcgill.ca).

## Abbreviations

AIDS: Acquired Immunodeficiency Syndrome
APRI: Aspartate Aminotransferase (AST) to platelet ratio
AUC: Area under the Receiver Operating Characteristic Curve
BMI: Body Mass Index
CCC: Canadian Co-infection Cohort
CD4: Cluster of differentiation 4 receptor
CDC: Centre for Disease Control
CES-D-10: Center for Epidemiologic Studies Depression Scale-10
CI: Confidence Interval
EQ-5D-3L: EuroQol-5 Dimension-3 Level
FS: Food Security
HCV: Hepatitis C virus
Hep B: Hepatitis B virus
HFSSM: Household Food Security Survey Module
HIV: Human Immunodeficiency Virus
HR-QoL: Health-related Quality of Life
IQR: Interquartile Range
LR-: Negative likelihood ratio
LR +: Positive likelihood ratio
NPV: Negative Predictive Value (NPV),
OOB: Out-of-Bag Samples
P6M: In the past 6 months
PPV: Positive Predictive Value
RF: Random Forests
RMSE: Root Mean Squared Error
RNA: Ribonucleic acid
ROC: Receiver Operating Characteristic
SES: Socioeconomic Status
