# Supplementary material for: Predicting the presence of depressive symptoms in the HIV-HCV co-infected population in Canada using supervised machine learning
Source: BMC Med Res Methodol. 2022 Aug 12;22:223. doi: 10.1186/s12874-022-01700-y (PMC9375382; doi:10.1186/s12874-022-01700-y)
Supplement: Supplementary file 1 — Additional file 1. [file 12874_2022_1700_MOESM1_ESM.docx]

**Supplement**

**Title:** Predicting the presence of depressive symptoms in the HIV-HCV co-infected population in Canada using supervised machine learning

**Appendix A:** Candidate predictors

**Appendix B:** Introduction to Random Forests

**Appendix C:** Supplementary methods and results

**Appendix D:** Sample generalizability

**Appendix A: Candidate Predictors**

Table S1 below provides an exhaustive list of predictors and their corresponding categories used in this analysis.

**Table S1: List of candidate predictors**

| **Sr. no.** | **Predictors** | **Categories** | **Participant visits (n=1934)**  **n (%) or Median (IQR)** | **Included in** | |
| --- | --- | --- | --- | --- | --- |
|  |  |  |  | **Reduced Algorithm**  **(x = 46)** | **Full**  **Algorithm**  **(x = 137)** |
| 1 | Have you ever been in a psychiatric institution or psychiatric hospital? (ever or since the last interview) | Yes  No  No response | 106 (5.5)  1825 (94.4)  3 (0.2) | Yes | Yes |
| 2 | Use of psychotropic medications | Yes  No | 884 (45.7)  1050 (54.3) | Yes | Yes |
|  | Has the patient ever had any of the following psychiatric diagnoses? (Ever at baseline) |  |  |  |  |
| 3 | Depression | Yes  No  No response | 102 (5.3)  1829 (94.6)  3 (0.2) | Yes | Yes |
| 4 | Bipolar disorder | Yes  No  No response | 12 (0.6)  1918 (99.2)  4 (0.2) | No | Yes |
| 5 | Schizophrenia | Yes  No  No response | 15 (0.8)  1915 (99.0)  4 (0.2) | No | Yes |
| 6 | Personality disorder | Yes  No  No response | 18 (0.9)  1912 (98.9)  4 (0.2) | No | Yes |
| 7 | Other psychiatric disorder | Yes  No | 40 (2.1)  1894 (97.9) | No | Yes |
| 8 | Any psychiatric diagnoses (other than depression) | Yes  No  No response | 69 (3.6)  1862 (96.3)  3 (0.2) | Yes | No |
|  | Use of specific substances |  |  |  |  |
| 9 | Cocaine | Yes  No | 553 (28.6)  1381 (71.4) | No | Yes |
| 10 | Crack | Yes  No | 373 (19.3)  1561 (80.7) | No | Yes |
| 11 | Heroin | Yes  No  No response | 216 (11.2)  1717 (88.8)  1 (0.1) | No | Yes |
| 12 | Speedball (Cocaine + Heroine) | Yes  No  No response | 34 (1.2)  1899 (98.2)  1 (0.1) | No | Yes |
| 13 | PCP/Mescaline | Yes  No  No response | 6 (0.3)  1927 (99.6)  1 (0.1) | No | Yes |
| 14 | Methadone | Yes  No | 125 (6.5)  1809 (93.5) | No | Yes |
| 15 | Morphine | Yes  No  No response | 125 (6.5)  1808 (93.4)  1 (0.1) | No | Yes |
| 16 | LSD | Yes  No  No response | 9 (0.5)  1924 (99.4)  1 (0.1) | No | Yes |
| 17 | Amphetamines | Yes  No  No response | 91 (4.7)  1842 (95.2)  1 (0.1) | No | Yes |
| 18 | Methamphetamine | Yes  No  No response | 152 (7.8)  1781 (92.1)  1 (0.1) | No | Yes |
| 19 | Talwin/Ritalin | Yes  No  No response | 8 (0.4)  1925 (99.5)  1 (0.1) | No | Yes |
| 20 | Ritalin alone | Yes  No  No response | 43 (2.2)  1890 (97.7)  1 (0.1) | No | Yes |
| 21 | Benzodiazepines | Yes  No | 86 (4.5)  1848 (95.5) | No | Yes |
| 22 | Barbiturates | Yes  No  No response | 16 (0.8)  1917 (99.1)  1 (0.1) | No | Yes |
| 23 | Dilaudid | Yes  No | 130 (6.7)  1804 (92.3) | No | Yes |
| 24 | Percocet | Yes  No  No response | 39 (2.0)  1894 (97.9)  1 (0.1) | No | Yes |
| 25 | Oxycodone | Yes  No  No response | 61 (3.2)  1872 (96.7)  1 (0.1) | No | Yes |
| 26 | Alcohol | Yes  No  No response | 13 (0.7)  1920 (99.2)  1 (0.1) | No | Yes |
| 27 | Freebase | Yes  No | 71 (3.7)  1863 (96.3) | No | Yes |
| 28 | Demerol | Yes  No | 3 (0.2)  1931 (99.8) | No | Yes |
| 29 | MDA | Yes  No | 17 (0.9)  1917 (99.1) | No | Yes |
| 30 | Mushrooms | Yes  No | 15 (0.8)  1919 (99.2) | No | Yes |
| 31 | Solvent - drink (Aqua Velva) | Yes  No | 4 (0.2)  1930 (99.8) | No | Yes |
| 32 | Solvent - sniff (gas, glue, Lysol, Pam) | Yes  No | 3 (0.2)  1931 (99.8) | No | Yes |
| 33 | Tylenol with codeine | Yes  No | 69 (3.6)  1865 (96.4) | No | Yes |
|  | EQ-5D- 3L instrument |  |  |  |  |
| 34 | Mobility | No problem in walking  Some problem in walking  Confined to bed  No response | 1319 (68.2)  605 (31.3)  8 (0.4)  2 (0.1) | No | Yes |
| 35 | Self-care | No problem with self-care  Some problem with self-care  Unable to wash or dress myself  No response | 1760 (91.0)  158 (8.2)  15 (0.8)  1 (0.1) | No | Yes |
| 36 | Usual activities | No problem in performing my usual activities  Some problem in performing my usual activities  Unable to perform my usual activities  No response | 1328 (68.7)  575 (29.8)  28 (1.5)  3 (0.2) | No | Yes |
| 37 | Pain and discomfort | No pain or discomfort  Moderate pain or discomfort  Extreme pain or discomfort  No response | 759 (39.3)  929 (48.0)  245 (12.7)  1 (0.1) | No | Yes |
| 38 | Anxiety and Depression | Not anxious or depressed  Moderately anxious or depressed  Extremely anxious or depressed  No response | 1002 (51.8)  772 (39.9)  157 (8.1)  3 (0.2) | No | Yes |
| 39 | Health State | Continuous; Range: 0 – 100; 0 = worst, 100 = best | 70 (60, 80) | No | Yes |
| 40 | Age | 15-25  26-35  36-45  46-55  56-65  66-75  >=75 | 3 (0.12)  147 (7.6)  387 (20.0)  944 (48.8)  414 (21.4)  36 (1.9)  3 (0.20 | Yes | Yes |
| 41 | Gender | Male  Female  Transgender No response | 1421 (73.5)  489 (25.3)  21 (1.1)  3 (0.2) | Yes | Yes |
| 42 | Marital status | Single  Married or common-law Widow(er) Divorced  No response | 1359 (70.3)  324 (16.8)  50 (2.6)  163 (8.4)  38 (1.9) | Yes | Yes |
|  | Race/ethnicity |  |  |  |  |
| 43 | Asian | Yes  No  No response | 26 (1.3)  1899 (98.2)  9 (0.5) | Yes | Yes |
| 44 | Black | Yes  No  No response | 75 (3.9)  1843 (95.3)  16 (0.8) | Yes | Yes |
| 45 | White | Yes  No  No response | 1483 (76.7)  437 (22.6)  14 (0.7) | Yes | Yes |
| 46 | Metis | Yes  No  No response | 80 (4.1)  1841 (95.2)  13 (0.7) | Yes | Yes |
| 47 | First Nation | Yes  No  No response | 260 (13.4)  1663 (86.0)  11 (0.6) | Yes | Yes |
| 48 | Hispanic/Latino | Yes  No  No response | 18 (0.9)  1907 (98.6)  9 (0.5) | Yes | Yes |
| 49 | Country of origin – Born in Canada | Yes  No  No response | 1470 (76.0)  175 (9.1)  289 (14.9) | Yes | Yes |
| 50 | Living situation | Fixed address  Share accommodations  Live in shelter or residence  Homeless  No response | 971 (50.2)  786 (40.6)      127 (6.6)  48 (2.5)  2 (0.1) | Yes | Yes |
| 51 | Education | Less than elementary  Elementary school  High school  College  University  No response | 16 (0.8)  348 (18.0)  1046 (54.1)  303 (15.7)  190 (9.8)  31 (1.6) | Yes | Yes |
| 52 | Employment | Not working for health reasons  Not working for lifestyle reasons  Not working but able to work  Studying  Part-time work  Full-time work  Homeworker  Retired  Other  No response | 1234 (63.8)  95 (4.9)  84 (4.3)  25 (1.3)  187 (9.7)  181 (9.4)  10 (0.5)  66 (3.4)  46 (2.4)  6 (0.3) | Yes | Yes |
| 53 | Monthly income | <= $1500  > $1500  No response | 1504 (77.8)  422 (21.9)  8 (0.4) | Yes | Yes |
| 54 | Revenue source | None  Welfare  Employment insurance  Disability insurance  Pension  Self-employment  Employment  Other  No response | 25 (1.3)  915 (47.3)  22 (1.1)  574 (29.7)  92 (4.8)  39 (2.0)  190 (9.8)  73 (3.8)  4 (0.2) | Yes | Yes |
| 55 | Shared accommodation – number of adults | 0  1-5  6-10  >10  No response | 362 (18.7)  751 (38.8)  29 (1.5)  23 (1.2)  769 (39.8) | No | Yes |
| 56 | Shared accommodation – number of children | 0  1-5  6-10  No response | 865 (44.7)  174 (9.0)  1 (0.1)  894 (46.2) | No | Yes |
| 57 | Sexual orientation | Heterosexual  Homosexual  Bisexual  No response | 1316 (68.1)  396 (20.5)  198 (10.2)  24 (1.2) | Yes | Yes |
| 58 | Current injection drug use | Yes  No | 627 (32.4)  1307 (67.6) | Yes | Yes |
| 59 | Current non-injection drug use | Yes  No | 633 (32.7)  1301 (67.3) | Yes | Yes |
| 60 | Current alcohol use | Yes  No | 1163 (60.1)  771 (39.9) | Yes | Yes |
| 61 | Alcohol abuse | Yes  No  Not applicable  No response | 313 (16.2)  874 (45.2)  739 (38.2)  8 (0.4) | Yes | Yes |
| 62 | Current smoking | Yes  No  No response | 1443 (74.6)  488 (25.2)  3 (0.2) | Yes | Yes |
| 63 | Been in jail | Yes  No  No response | 1203 (62.2)  726 (37.5)  5 (0.3) | Yes | Yes |
| 64 | Therapy or in a program for drug addiction | Yes  No  No response | 1233 (63.8)  649 (33.6)  52 (2.6) | No | Yes |
| 65 | Therapy or in a program for drug addiction (last 6 months) | Yes  No  No response | 385 (19.9)  1497 (77.4)  52 (2.7) | No | Yes |
| 66 | Therapy or in a program for alcohol addiction | Yes  No  Not applicable  No response | 544 (28.1)  1220 (63.1)  2 (0.1)  168 (8.7) | No | Yes |
| 67 | Marijuana | Yes  No  No response | 1039 (53.7)  892 (46.1)  3 (0.2) | Yes | Yes |
| 68 | Frequency of Marijuana use | Not applicable (if don’t use pot)  Occasionally - not every week  Regularly - 1-2 days per week  Regularly - 3-6 days per week  Everyday  No response | 892 (46.1)  296 (15.3)  142 (7.3)  149 (7.7)  452 (23.4)  3 (0.2) | No | Yes |
| 69 | Why do you use Marijuana? | Not applicable  Relieve symptoms  Increase appetite  Fun  Symptoms and appetite  Symptoms and fun  Appetite and fun  All three  No response | 893 (46.2)  270 (14.0)  229 (11.8)  333 (17.2)  90 (4.7)  26 (1.3)  17 (0.9)  37 (1.9)  39 (2.0) | No | Yes |
| 70 | Engaged in sex work in the past 6 months | Yes  No  No response | 88 (4.6)  1832 (94.7)  14 (0.7) | No | Yes |
| 71 | Used services of sex workers in the past 6 months | Yes  No  No response | 61 (3.2)  1860 (96.2)  13 (0.7) | No | Yes |
| 72 | Body piercing | Yes  No  No response | 122 (6.3)  1808 (93.5)  4 (0.2) | No | Yes |
| 73 | Tattoo | Yes  No  No response | 189 (9.8)  1735 (89.7)  10 (0.5) | No | Yes |
| 74 | IDU ever | Yes  No  No response | 1616 (83.6)  308 (15.9)  10 (0.5) | Yes | Yes |
| 75 | Shared needles ever if IDU=1 | Yes  No  Not applicable  No response | 1191 (61.6)  421 (21.8)  280 (14.5)  42 (2.2) | No | Yes |
| 76 | Equipment share ever if IDU=1 | Yes  No  Not applicable  No response | 1151 (59.5)  470 (24.3)  282 (14.6)  31 (1.6) | No | Yes |
| 77 | Injected Drugs in jail ever if IDU=1 | Yes  No  Never been in jail  Not applicable  No response | 269 (13.9)  1023 (52.9)  317 (16.4)  289 (14.9)  36 (1.9) | No | Yes |
| 78 | Snort ever | Yes  No  No response | 1609 (83.2)  282 (14.6)  43 (2.2) | Yes | Yes |
| 79 | Snort share ever | Yes  No  Not applicable  No response | 1177 (60.9)  552 (28.5)  144 (7.5)  61 (3.2) | No | Yes |
| 80 | Shared needles in the past 6 months if IDU_6m=1 | Yes  No  Not applicable  No response | 38 (2.0)  602 (31.3)  1145 (59.2)  149 (7.7) | No | Yes |
| 81 | Equipment share in the past 6 months if IDU_6m=1 | Yes  No  Not applicable  No response | 62 (3.2)  705 (36.5)  1119 (57.9)  48 (2.5) | No | Yes |
| 82 | Injected Drugs in jail in the past 6 months if IDU_6m=1 | Yes  No  Not been in jail in the past 6 months  Not applicable  No response | 9 (0.5)  225 (11.6)  921 (47.6)  773 (39.9)  6 (0.3) | No | Yes |
| 83 | Snort in the past 6 months | Yes  No  No response | 425 (21.9)  1502 (77.7)  7 (0.4) | No | Yes |
| 84 | Snort share in the past 6 months | Yes  No  Not applicable  No response | 103 (5.3)  1224 (63.3)  560 (28.9)  47 (2.4) | No | Yes |
| 85 | Did you get used equipment in the past 6 months? | Yes  No  Not applicable  No response | 79 (4.1)  668 (34.5)  1139 (58.9)  48 (2.5) | No | Yes |
| 86 | Did you give used equipment in the past 6 months? | Yes  No  Not applicable  No response | 55 (2.8)  831 (42.9)  989 (51.1)  59 (3.1) | No | Yes |
| 87 | Ever smoked | Yes  No  No response | 1738 (89.9)  175 (9.1)  21 (1.1) | Yes | Yes |
| 88 | Previously consumed alcohol | Yes  No  Not applicable  No response | 767 (39.7)  166 (8.6)  992 (51.3)  9 (0.5) | Yes | Yes |
| 89 | No. of male sexual partners ever had in life | 0  1  2-5  6-10  11-50  51-100  >100  No response | 769 (39.8)  41 (2.1)  162 (8.4)  164 (8.5)  219 (11.3)  140 (7.2)  401 (20.7)  38 (2.0) | No | Yes |
| 90 | No. of female sexual partners ever had in life | 0  1  2-5  6-10  11-50  51-100  >100  No response | 507 (26.2)  115 (6.0)  359 (18.6)  255 (13.2)  409 (21.2)  138 (7.1)  116 (6.0)  35 (1.8) | No | Yes |
| 91 | No. of male sexual partners in the past 6 months | 0  1  2-5  6-10  11-50  51-100  >100  No response | 1332 (68.9)  332 (17.2)  169 (8.7)  47 (2.4)  23 (1.2)  6 (0.3)  12 (0.6)  13 (0.7) | No | Yes |
| 92 | No. of female sexual partners in the past 6 months | 0  1  2-5  6-10  11-50  51-100  >100  No response | 1457 (75.3)  338 (17.5)  83 (4.3)  15 (0.8)  16 (0.8)  7 (0.4)  5 (0.3)  13 (0.7) | No | Yes |
| 93 | Shot up with steady sex partner in the past 6 months | Yes  No  Not applicable  No response | 113 (5.8)  370 (19.1)  1294 (66.9)  157 (8.1) | No | Yes |
| 94 | Shot up with close friend or family member in the past 6 months | Yes  No  Not applicable  No response | 135 (6.9)  351 (18.2)  1296 (67.0)  152 (7.9) | No | Yes |
| 95 | Shot up with people the patient doesn’t know very well in the past 6 months | Yes  No  Not applicable  No response | 63 (3.3)  407 (21.0)  1296 (67.0)  168 (8.7) | No | Yes |
| 96 | Shot up with people the patient doesn’t know at all in the past 6 months | Yes  No  Not applicable  No response | 26 (1.3)  434 (22.4)  1296 (67.0)  178 (9.2) | No | Yes |
| 97 | Shot up with nobody (alone) in the past 6 months | Yes  No  Not applicable  No response | 353 (18.3)  262 (13.6)  1311 (67.8)  8 (0.4) | No | Yes |
| 98 | Got needles or injecting equipment from steady sex partner in the past 6 months | Yes  No  Not applicable  No response | 24 (1.2)  9 (0.5)  1799 (93.0)  102 (5.3) | No | Yes |
| 99 | Got needles or injecting equipment from close friend or family member in the past 6 months | Yes  No  Not applicable  No response | 24 (1.2)  11 (0.6)  1799 (93.0)  100 (5.2) | No | Yes |
| 100 | Got needles or injecting equipment from people the patient doesn’t know very well in the past 6 months | Yes  No  Not applicable  No response | 22 (1.1)  10 (0.5)  1800 (93.1)  102 (5.3) | No | Yes |
| 101 | Got needles or injecting equipment from people the patient doesn’t know at all in the past 6 months | Yes  No  Not applicable  No response | 6 (0.3)  15 (0.80)  1800 (93.1)  113 (5.8) | No | Yes |
| 102 | Got needles or injecting equipment from nobody in the past 6 months | No  Not applicable  No response | 15 (0.8)  1800 (93.1)  99 (6.2) | No | Yes |
| 103 | Give needles or injecting equipment to steady sex partner in the past 6 months | Yes  No  Not applicable  No response | 23 (1.2)  3 (0.2)  1833 (94.8)  75 (3.9) | No | Yes |
| 104 | Give needles or injecting equipment to close friend or family member in the past 6 months | Yes  No  Not applicable  No response | 16 (0.8)  14 (0.7)  1829 (94.6)  75 (3.9) | No | Yes |
| 105 | Give needles or injecting equipment to people the patient doesn’t know very well in the past 6 months | Yes  No  Not applicable  No response | 9 (0.5)  7 (0.4)  1815 (93.9)  103 (5.3) | No | Yes |
| 106 | Give needles or injecting equipment to people the patient doesn’t know at all in the past 6 months | Yes  No  Not applicable  No response | 8 (0.4)  8 (0.4)  1815 (93.9)  103 (5.3) | No | Yes |
| 107 | Give needles or injecting equipment to nobody (alone) in the past 6 months | No  Not applicable No response | 8 (0.4)  1816 (93.9)  110 (5.7) | No | Yes |
| 108 | BMI | Underweight  Normal weight  Overweight  Obese  No response | 117 (6.1)  860 (44.5)  479 (24.8)  238 (12.3)  240 (12.4) | Yes | Yes |
| 109 | HIV Viral load | <= 50 (undetectable)  > 50  No response | 1591 (82.3)  316 (16.3)  27 (1.4) | Yes | Yes |
| 110 | CD4 count | <= 250 (low)  > 250  No response | 323 (16.7)  1599 (82.7)  12 (0.6) | Yes | Yes |
| 111 | HCV RNA status | Detectable  Not detectable  Not done | 815 (42.1)  383 (19.8)  736 (38.1) | Yes | Yes |
| 112 | HIV disease stage | A1  A2  A3  B1  B2  B3  C1  C2  C3  No response | 719 (37.2)  566 (29.3)  168 (8.7)  63 (3.3)  45 (2.3)  11 (0.6)  28 (1.5)  35 (1.8)  38 (2.0)  261 (13.5) | Yes | Yes |
| 113 | Sexually transmitted disease (STD) in the past 6 months | Yes  No  No response | 73 (3.8)  1843 (95.3)  18 (0.9) | Yes | Yes |
| 114 | Ever diagnosed with hepatitis B | Yes  No  Unknown  No response | 364 (18.8)  1184 (61.2)  336 (17.4)  50 (2.6) | No | Yes |
| 115 | Cirrhosis | Yes  No | 46 (2.4)  1888 (97.6) | Yes | Yes |
| 116 | Ascites | Yes  No | 5 (0.3)  1929 (99.7) | Yes | Yes |
| 117 | Varices | Yes  No | 7 (0.4)  1927 (99.6) | Yes | Yes |
| 118 | Portal hypertension | Yes  No | 6 (0.3)  1928 (99.7) | Yes | Yes |
| 119 | Encephalopathy | Yes  No | 1 (0.1)  1933 (99.9) | Yes | Yes |
| 120 | Hepatocellular carcinoma | Yes  No | 2 (0.1)  1932 (99.9) | Yes | Yes |
| 121 | AIDS defining illness | Yes  No  No response | 38 (2.0)  1885 (97.5)  11 (0.6) | Yes | Yes |
| 122 | Cardiovascular disease | Yes  No  No response | 27 (1.4)  1906 (98.5)  1 (0.1) | No | Yes |
| 123 | Hypercholesterolemia | Yes  No  No response | 22 (1.1)  1911 (98.8)  1 (0.1) | No | Yes |
| 124 | Autoimmune disease | Yes  No  No response | 4 (0.2)  1928 (99.7)  2 (0.1) | No | Yes |
| 125 | Hypertension | Yes  No  No response | 47 (2.4)  1885 (97.5)  2 (0.10 | No | Yes |
| 126 | Thyroid disease | Yes  No  No response | 9 (0.5)  1922 (99.4)  3 (0.2) | No | Yes |
| 127 | Lipodystrophy | Yes  No  No response | 12 (0.6)  1916 (99.1)  6 (0.3) | No | Yes |
| 128 | Psoriasis | Yes  No  No response | 15 (0.8)  1913 (98.9)  6 (0.3) | No | Yes |
| 129 | Diabetes | Yes  No  No response | 15 (0.8)  1914 (98.9)  5 (0.3) | No | Yes |
| 130 | Previous Interferon-based HCV treatment | Yes  No  No response | 249 (12.9)  1671 (86.4)  14 (0.7) | Yes | Yes |
|  | In the past 6 months, how many times did you visit the following health services? |  |  |  |  |
| 131 | Walk-in clinic | 0  1-10  11-20  21-30  >30  No response | 1588 (82.1)  325 (16.8)  11 (0.6)  2 (0.1)  2 (0.1)  6 (0.3) | No | Yes |
| 132 | Emergency room | 0  1-10  11-20  21-30  No response | 1377 (71.2)  537 (27.8)  9 (0.5)  1 (0.1)  10 (0.5) | No | Yes |
| 133 | Hospital inpatient – overnight | 0  1-10  11-20  21-30  >30  No response | 1616 (83.6)  260 (13.44)  23 (1.2)  14 (0.7)  11 (0.6)  10 (0.5) | No | Yes |
| 134 | General practitioner | 0  1-10  11-20  21-30  >30  No response | 1176 (60.8)  666 (34.4)  70 (3.6)  9 (0.5)  5 (0.3)  8 (0.4) | No | Yes |
| 135 | HIV clinic | 0  1-10  11-20  21-30  >30  No response | 508 (26.3)  1328 (68.7)  78 (4.0)  12 (0.6)  3 (0.2)  5 (0.3) | No | Yes |
| 136 | A specialist | 0  1-10  11-20  21-30  >30  No response | 1231 (63.7)  666 (34.4)  14 (0.70  7 (0.4)  2 (0.1)  14 (0.7) | No | Yes |
| 137 | APRI for significant liver fibrosis | APRI <1.5  APRI > 1.5  No response | 304 (15.7)  1509 (78.0)  121 (6.3) | No | Yes |
| 138 | Currently on ARV | Yes  No | 1809 (93.5)  125 (6.5) | Yes | Yes |
| 139 | Food insecurity using the Household Food Security Survey Module (HFSSM)^*^ | Food secure  Moderately food insecure  Severely food insecure | 906 (46.9)  424 (21.9)  604 (31.2) | No | No |
|  |  | **Total number of predictors** |  | **46** | **137** |

* Used only in the additional analysis.

**Abbreviations:** IQR: Interquartile range; PCP: Phenylcyclohexyl piperidine; LSD: Lysergic acid diethylamide; MDA: Methylenedioxyamphetamine; EQ-5D-3L: EuroQoL-5Dimension-3Level; IDU: Injection drug use; BMI: Body Mass Index; HIV: Human Immunodeficiency Virus; CD4: Cluster of differentiation 4 receptor; HCV: Hepatitis C virus; RNA: Ribonucleic acid; AIDS: Acquired Immunodeficiency Syndrome; APRI: Aspartate Aminotransferase (AST) to platelet ratio

**Appendix B: Introduction to Random Forests**

We used the supervised machine learning technique of random forests (RF), an ensemble learning technique developed by Leo Breiman [1, 2]. Ensemble learning is based on the idea of combining the strengths of many simpler “base” models. The base models in the case of RF are decision trees, specifically classification and regression trees (CART) for binary and continuous outcomes, respectively.[3] RF classification uses bootstrap aggregation of multiple decision trees, combining the predictions from these many trees. A decision tree is made up of nodes (root node, decision node and terminal nodes) and branches. Each node represents a predictor variable, which is chosen from a random subset of all predictor variables, which is a characteristic of the RF algorithm.[3] Multiple decision trees are generated from bootstrapped samples of the training data, which is usually about a 2/3 subset of the data. The test set, which is the remaining data, is then run through these trees and the response estimate is the average over all the individual predictions in the forest.[1-5]

The main characteristics of RF are:

**1) Ensemble technique** with multiple decision trees help reduce overfitting [3];

**2)** **Selection of a random subset of predictors from all candidates at each node** of which one predictor that most improves accuracy is then selected; the random selection makes each tree more independent of the other and reducing correlation [3, 4];

**3)** **Out-of-Bag (OOB) samples** are the observations that are not selected in a given bootstrap resample and using these samples, OOB error is calculated as the average discrepancy between actual outcome and the outcome predicted by the RF, which provides an additional internal validation.[3, 4]

When developing the RF, the parameters of the algorithm are tuned, i.e., a set of parameters are selected which minimize the OOB error and thus maximizing accuracy. These tuning parameters include the number of trees (B), the number of predictors chosen from all candidate predictors at each node (m), and three characteristics of tree depth: node size (s), which is maximum number of observations at each node, maximum terminal nodes (u), and tree level (k), which is maximum number of splits.[6]

The algorithm also provides importance metrics to provide an idea which variables were the most influential in the classification algorithm. Overall, RFs are non-parametric, accurate, and relatively robust to outliers and noise. RF variable importance graphs provide insights regarding which variables play a major role in the accurate prediction. Regression RF algorithms can be used to predict for continuous outcomes and classification algorithms can be used to classify into binary or categorical outcomes. Additionally, probability machines for the classification can be used to estimate the probabilities for class membership, which can then be used to determine the class at the optimal probability thresholds.[7]

**Appendix C: Supplementary methods and results**

**RF tuning**

The RF hyperparameters used to tune the RF algorithms to maximize accuracy were as follows:

- Number of trees (B): Range used: 50-1500
- Number of predictors, randomly chosen from all predictors in the algorithm at each node (mtry): Range used: sqrt(x) to x/2), where x= total number of candidate predictors
- Tree depth, which is maximum number of splits (D): Range used: 1-30 and 0 = no limit
- Node size, which is minimum number of observations at each node (S): Range used: 1-21

The ranges for the hyperparameters were selected based on recommendations in literature [6, 8].

Using the training data, we conducted 10-fold cross validation using grids of the above hyperparameter ranges, the final parameters which maximized accuracy were chosen and these are given in Table C.1 for all algorithms.

**Overall performance**

The overall performance was evaluated using a scaled Brier score (BS_scaled_), which is the Brier score (BS) or mean squared error scaled with the maximum mean squared error in a model that randomly classifies into either CES-D-10 class (BS_max_): BS_scaled_=1-(BS/BS_max_).[9] Relative efficiency was calculated by comparing the BS_scaled_ for the two algorithms [10, 11].

In the testing data, the full algorithm had a BS_scaled_ of 0.31 (95% CI: 0.22-0.39), that is a 31% reduction in mean squared error as compared to random classification and the reduced algorithm had a BS_scaled_ of 0.20 (95% CI: 0.12-0.27), which means a 20% reduction [10]. The relative efficiency comparing the reduced to the full algorithm was 0.65 (95% CI: 0.38-1.01), i.e., a 65% loss in efficiency, indicating better performance of the full algorithm compared to reduced algorithm.

**Calibration**

Calibration is assessment of the agreement between observed and predicted outcomes. We generated calibration graphs by plotting the predicted probabilities for being in CES-D-10 class=1 (CES-D-10 score >=10) against the observed frequency of CES-D-10 class=1 [11]. The Stata package pmcalplot was used to create the calibration graph [12]. The calibration graphs are shown in Figure C.1.

The calibration measure, calibration slope was estimated, with bootstrapped 95% confidence intervals (CI). For the full algorithm, the calibration slope measure was 0.95 (95% CI: 0.72-1.21) for the reduced algorithm and this estimate was close to the ideal calibration slope of 1 for a well-calibrated algorithm [11]. Similarly, For the reduced algorithm, the calibration slope measure was 0.97 (95% CI: 0.72-1.21) and this estimate was close to the ideal calibration slope.

**Table S2**: Final RF hyperparameters for the algorithms

| **Sr. No.** | | **Algorithms** | **OOB error** | **B** | **mtry** | **D** | **S** |
| --- | --- | --- | --- | --- | --- | --- | --- |
| **Primary analysis** | | | | | | | |
| 1 | Full algorithm (x = 137) | | 0.16 | 600 | 70 | 0 (no limit) | 1 |
| 2 | Reduced algorithm (x=46) | | 0.20 | 800 | 20 | 0 (no limit) | 1 |
| **Additional analyses** | | | | | | | |
| **A** | | **One visit per individual (x=46)** |  |  |  |  |  |
| 1 | | Full algorithm (x = 137) | 0.17 | 1000 | 80 | 10 | 1 |
| 2 | | Reduced algorithm (x=46) | 0.23 | 100 | 15 | 10 | 5 |
| **B** | | **Different CES-D-10 cut-offs** | | | | | |
| **I** | | **Cut-off - 8** | | | | | |
| 1 | | Full algorithm (x = 137) | 0.17 | 100 | 70 | 0 (no limit) | 5 |
| 2 | | Reduced algorithm (x=46) | 0.19 | 400 | 10 | 0 (no limit) | 1 |
| **II** | | **Cut-off - 13** | | | | | |
| 1 | | Full algorithm (x = 137) | 0.15 | 1400 | 50 | 0 (no limit) | 1 |
| 2 | | Reduced algorithm (x=46) | 0.20 | 1200 | 20 | 0 (no limit) | 1 |
| **III** | | **Cut-off - 15** | | | | | |
| 1 | | Full algorithm (x = 137) | 0.14 | 200 | 30 | 0 (no limit) | 1 |
| 2 | | Reduced algorithm (x=46) | 0.17 | 100 | 10 | 20 | 1 |
| **C** | | **With food insecurity variable (x=138)** | 0.16 | 100 | 80 | 10 | 1 |
| **D** | | **Regression algorithm** | | | | | |
| 1 | | Full algorithm (x = 137) | 0.24 | 1400 | 80 | 0 (no limit) | 1 |
| 2 | | Reduced algorithm (x=46) | 0.35 | 200 | 15 | 0 (no limit) | 1 |

**Abbreviations:** OOB: Out-Of-Bag sample; B: Number of trees; mtry: Number of predictors chosen randomly at each node; D: tree depth; S: minimum node size; CES-D-10: Center for Epidemiologic Studies Depression Scale-10; EQ-5D-3L: EuroQoL-5Dimension-3Level

**Figure S1:** Calibration graphs - A. Full algorithm (x = 137) and B. Reduced algorithm (x = 46)

B. Reduced Algorithm (x=46)

A. Full Algorithm (x=137)

**Abbreviations:** AUC: Area under the Receiver Operating Characteristic curve

**Appendix D: Sample generalizability**

**Table S3:** Participant characteristics in visit 1 in the study sample (n=717) as compared to baseline visit for all CCC participants (n=2008)

| **Characteristics** | **Participants (n = 717)**  **n (%) or median (IQR)** | **Participants (n=2008)**  **n (%) or median (IQR)** |
| --- | --- | --- |
| Age | 49 (43, 54) | 45 (39, 52) |
| Gender - Male | 522 (73) | 1412 (70) |
| Race/Ethnicity |  |  |
| Asian | 11 (2) | 36 (2) |
| Black | 28 (4) | 70 (4) |
| White | 541 (76) | 1401 (70) |
| Metis | 32 (5) | 102 (5) |
| First nation | 102 (14) | 412 (21) |
| Hispanic/Latino | 7 (1) | 29 (1) |
| Born outside Canada | 64 (9) | 181 (9) |
| Education - High school and higher | 565 (79) | 1532 (76) |
| Employment - Not employed | 525 (73) | 1334 (66) |
| Monthly income  $1500 | 543 (76) | 1526 (76) |
| Revenue Source - Welfare | 332 (46) | 954 (48) |
| Current injection drug use | 244 (34) | 819 (41) |
| Current alcohol use | 444 (62) | 950 (47) |
| Current smoking | 534 (75) | 1505 (75) |
| BMI category - Normal weight (18.5-25 kg/m^2^) | 312 (44) | 798 (40) |
| End-stage Liver disease | 27 (4) | 57 (3) |
| HIV clinical stage - A1 (asymptomatic) | 248 (35) | 438 (22) |
| Past AIDS related illness | 28 (4) | 458 (23) |
| Depression diagnosis | 68 (10) | 742 (37) |
| Prescribed antidepressant medications | 320 (45) | 607 (30) |
| HR-QoL using EQ-5D-3L instrument  Anxiety/depression  Not anxious or depressed  Moderately anxious or depressed  Extremely anxious or depressed  Current health state (visual analog scale) | 352 (49)  302 (42)  60 (8)  70 (56, 80) | 747 (37)  818 (41)  183 (9)  70 (50, 80) |

**Abbreviations:** IQR: Interquartile range; AIDS: Acquired Immunodeficiency Syndrome; HIV: Human Immunodeficiency Virus; BMI: Body Mass Index; HR-QoL: Health related quality of life; EQ-5D-3L: EuroQoL-5Dimension-3Level

**References**

1. Breiman L. Random Forests. Machine Learning. 2001;45(1):5-32.

2. Fawagreh K, Gaber MM, Elyan E. Random forests: from early developments to recent advancements AU - Fawagreh, Khaled. Systems Science & Control Engineering. 2014;2(1):602-9.

3. Hastie T, Tibshirani R, Friedman JH. The elements of statistical learning : data mining, inference, and prediction. Second edition. ed. New York: Springer; 2009.

4. Mennitt D, Sherrill K, Fristrup K. A geospatial model of ambient sound pressure levels in the contiguous United States. The Journal of the Acoustical Society of America. 2014;135(5):2746-64.

5. Strobl C, Malley J, Tutz G. An introduction to recursive partitioning: rationale, application, and characteristics of classification and regression trees, bagging, and random forests. Psychological methods. 2009;14(4):323-48.

6. Scornet E, Coeurjolly J-Fo, Leclercq-Samson A. Tuning parameters in random forests. ESAIM: Proceedings and Surveys. 2017;60:144-62.

7. Malley JD, Kruppa J, Dasgupta A, Malley KG, Ziegler A. Probability machines: consistent probability estimation using nonparametric learning machines. Methods Inf Med. 2012;51(1):74-81.

8. Touw WG, Bayjanov JR, Overmars L, Backus L, Boekhorst J, Wels M, et al. Data mining in the Life Sciences with Random Forest: a walk in the park or lost in the jungle? Briefings in bioinformatics. 2013;14(3):315-26.

9. Steyerberg EW, Vickers AJ, Cook NR, Gerds T, Gonen M, Obuchowski N, et al. Assessing the performance of prediction models: a framework for traditional and novel measures. Epidemiology. 2010;21(1):128-38.

10. Wong J, Manderson T, Abrahamowicz M, Buckeridge DL, Tamblyn R. Can Hyperparameter Tuning Improve the Performance of a Super Learner?: A Case Study. Epidemiology. 2019;30(4):521-31.

11. Steyerberg EW. Clinical prediction models : a practical approach to development, validation, and updating. Cham, Switzerland: Springer; 2019.

12. Joie E, Kym IES, Emma CM. PMCALPLOT: Stata module to produce calibration plot of prediction model performance. S458486 ed: Boston College Department of Economics; 2018.
